# Seronegative coeliac disease: Are they coeliac? When biopsy in adult can be avoided? 

**Published:** 2018

**Authors:** Kamran Rostami, David Aldulaimi, Chris J Mulder

**Affiliations:** 1 *Department of Gastroenterology, Milton Keynes University Hospital, Milton Keynes, UK. *; 2 *Department of Gastroenterology, South Warwickshire Foundation Trust, Warwick, UK*; 3 *Department of Gastroenterology, VU University Medical Center Amsterdam *

## Introduction

Recent studies by the Derby team on the value of serology and narrowing the indications for small bowel biopsy generated a significant interest and opened a new chapter in improving the diagnostic policies for Celiac Disease (CD) in particular for children (1, 2). In this issue of GHFBB (3), the authors claim the new policy will not only simplify the diagnosis of CD for affected individuals, but also it has significant safety implications and cost reduction for health care systems. The novel new algorithm is particularly important for the young children who require general anaesthesia for their gastroscopy and duodenal biopsies, in keeping with recommendations from the European Society of Paediatric Gastroenterology and Nutrition (ESPGHAN), and it is incorporated in ESPGHAN guidelines (2, 4). 

The "biopsy-sparing" protocol seems to be applicable to both symptomatic and asymptomatic patients (5). Appropriately used serological tests may confer similar, if not higher, sensitivity and specificity for CD diagnosis than traditional duodenal biopsies. The use of serology for diagnosis, rather than screening, would change the current diagnostic paradigm for CD. However, using serological tests for diagnosis, and not relying on duodenal biopsies, has limitations. Most importantly, the availability of several serological kits that have a variable sensitivity and specificity and the lack of kit standardisation may decrease the accuracy of diagnosis of CD. The other main concern relates to avoiding endoscopy and biopsies. Avoiding endoscopy could lead to missing significant concurrent upper gastrointestinal pathology, such as stomach ulceration. Duodenal biopsies enable diagnosis of serology negative CD and other small bowel diseases, such as tropical sprue and Crohn’s disease. Furthermore, some studies based on endoscopy suggest a lower positive predictive value for serology than for endoscopy with duodenal biopsies (6). It is there important to consider both the advantages and limitations of a ‘biopsy sparing’ approach.

**Comparison histology and serology**

It is now rare for CD to present with sever malabsorption and classical histology with complete villous flattening. Over the last few decades, gastroenterologists and primary care physicians have been able to identify patients with non-specific symptoms, such as bloating and a milder enteropathy, helped by the availability of serological tests. CD can now be diagnosed in the presence of a mild enteropathy, with the benefit of sensitive and specific serological markers. These serological markers increase the higher sensitivity and specificity for diagnosing CD in the presence of mild enteropathy. Unfortunately, evidence of a milder enteropathy is often ignored and considered non-specific, rather than investigated (7). In addition, introduction of a gluten free diet (GFD) can also aid diagnosis, if repeat biopsies are performed (8). It is also clear that a small bowel enteropathy is not specific for CD, and the differential diagnosis of non-coeliac villous flattening continues to increase (9). See table 1.

**Table 1:** Differential diagnosis for small bowel architectural distortion

Coeliac disease
Seronegative gluten sensitives
Autoimmune enteropathy
Common variable immune deficiency
Collagenous sprue
Drug induced: *Rituximab enteropathy * *Sartan enteropathy*
Giardia lamblia
Eosinofiele enteropathy
Tropical sprue

**Seronegative coeliac disease**

**Figure 1:**
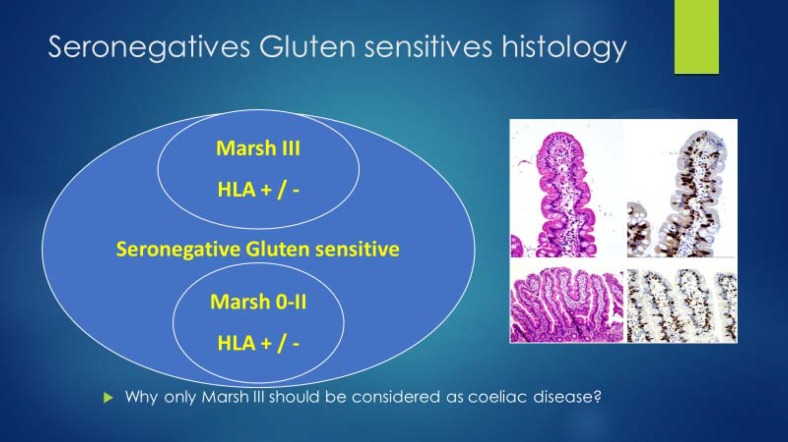
Gluten sensitive seronegatives with Marsh III should be classified in the same category as gluten sensitive seronegatives with Marsh 0-II

Seronegative CD was reported for the first time in 1998 (10, 11). This CD patient subgroup was reported in the era when non-coeliac gluten sensitivity (NCGS) was not recognised or properly defined. The diagnosis of seronegative CD was dependant on an enteropathy and a response to GFD. Later HLA typing was used to confirm the diagnosis. However, HLA has a very poor positive predictive value. The issue of seronegative CD has been very controversial, and the condition is considered to be rare, (12) or transient. Those cases with persistent symptoms in our opinion should be defined under NCGS. In NCGS, HLA does not add much and seems unreliable in solo for a diagnosis of CD (Figure 1).

It is, however, important to realize that both milder and severe enteropathies can be non-specific and there is a wide differential diagnosis (7, 9) Table 1. We suggest that in the absence of a positive tTG EMA, a diagnosis of NCGS should be considered in gluten sensitive cases, regardless to the degree of histological abnormality. Recognising that a severe enteropathy can develop with NCGS will provide greater insight in the patho-mechanism of NCGS.

**The value of small bowel biopsy in follow up**

According to several studies Recovery of small intestinal mucosal abnormalities are very slow and is incomplete or absent in a substantial subgroup of patients. Approximately, 50% of patients will still have persistent villous flattening in 12 months after initial biopsies,. Furthermore, 6-10% of patients will never achieve mucosal healing according (13, 14). This would suggest that routine small bowel biopsy is not beneficial for a significant proportion of patients diagnosed with CD. We suggest a follow up biopsy should be reserved for cases at high risk of complications and persistent signs of malabsorption syndrome. Refractory CD is a very rare condition and will undergo a follow up biopsy as they are very ill and belong to the high risk group. A clear criteria for this subgroup should be implemented for follow up biopsy in this group even though RCD 2 is very rare (15, 16).

The policy of avoiding endoscopy and biopsy in low risk children with a tTG of 8-10x/UNV might be safe and practical. It is likely that a significant proportion of young, and some older adult patients, can also safely avoid endoscopy and biopsy for initial diagnosis and during their follow up. 

However, there will be a need for a clear guideline, for instance from the European Society for the Study of Celiac Disease, to ensure that avoiding endoscopy and biopsy will not compromise the high quality care. 

## Conflict of interests

The authors declare that they have no conflict of interest.
